# Supplementary material for: Rural and urban differences in the prevalence and determinants of Type-2 diabetes in Bangladesh
Source: PLoS One. 2024 Apr 11;19(4):e0298071. doi: 10.1371/journal.pone.0298071 (PMC11008877; doi:10.1371/journal.pone.0298071)
Supplement: Attachment — Submitted filename: PONE-D-23-16040.pdf [file pone.0298071.s001.pdf]

PLOS ONE

Rural and Urban Differences in the Prevalence of Type-2 Diabetes and the Associated Risk Factors: Analysis of a Population-Based Nation-wide Cross-sectional Survey in Bangladesh

--Manuscript Draft--

Manuscript Number:	PONE-D-23-16040
Article Type:	Research Article
Full Title:	Rural and Urban Differences in the Prevalence of Type-2 Diabetes and the Associated Risk Factors: Analysis of a Population-Based Nation-wide Cross-sectional Survey in Bangladesh
Short Title:	Rural and Urban Differences in the Prevalence of Type-2 Diabetes
Corresponding Author:	Ashis Talukder<br>Khulna University<br>Khulna, BANGLADESH
Keywords:	Type 2 Diabetes; Urban; Rural; Risk factors; Cross-sectional Survey; Bangladesh
Abstract:	Objective: To estimate the prevalence of T2D in both urban and rural settings and identify the risk factors specific to each location.<br>Method: We explore the Bangladesh Demographic and Health Survey 2017-18 data sourcing from DHS website. A stratified two-stage sample was used for this survey where 7658 women and 7048 men aged 18 and older had found their blood glucose levels measured. Chi-square test and ordinal logistic regression were used to find the association between selected variables of both urban and rural settings with diabetes and prediabetes.<br>Results: The prevalence of diabetes is 10.8% and 7.4%, and pre-diabetes is 31.4% and 27% in urban and rural areas, respectively. The study identifies significant influences on diabetes of 55-64 age group in both urban and rural settings (Urban: AOR= 1.882, CI [1.462, 2.421]; Rural: AOR=1.874, CI [1.545,2.273]). The odds of diabetes were lower among highly educated participants, and higher among rich and overweight participants in both areas. In rural areas, the odds of diabetes is higher among caffeinators and physically inactive participants, whilst those show insignificant influence in urban areas. Moreover, the participants from urban areas show a considerable association between diabetes and hypertension.<br>Conclusion: Our findings urge the importance of location specific imminent preventive measures for T2D and prediabetes considering the concentration of the socio-demographic risk factors in both urban and rural contexts which essentially demand a sound public health policy to prevent the looming threat of public health sector.
Order of Authors:	Ashis Talukder
	Sabiha Shirin Sara
	Md. Tanvir Hossain
	Chuton Deb Nath
	Riaz Rahman
	Sadiq Hussain
	Md. Nazmul Huda
Additional Information:	
Question	Response
**Financial Disclosure**<br><br>Enter a financial disclosure statement that describes the sources of funding for the work included in this submission. Review	NO

the submission guidelines for detailed requirements. View published research articles from *PLOS ONE* for specific examples.<br><br>This statement is required for submission and **will appear in the published article** if the submission is accepted. Please make sure it is accurate.<br><br>**Unfunded studies**<br>Enter: *The author(s) received no specific funding for this work.*<br><br>**Funded studies**<br>Enter a statement with the following details:<br>• Initials of the authors who received each award<br>• Grant numbers awarded to each author<br>• The full name of each funder<br>• URL of each funder website<br>• Did the sponsors or funders play any role in the study design, data collection and analysis, decision to publish, or preparation of the manuscript?<br>• **NO** - Include this sentence at the end of your statement: *The funders had no role in study design, data collection and analysis, decision to publish, or preparation of the manuscript.*<br>• **YES** - Specify the role(s) played.<br><br>* typeset	
**Competing Interests**<br><br>Use the instructions below to enter a competing interest statement for this submission. On behalf of all authors, disclose any competing interests that could be perceived to bias this work—acknowledging all financial support and any other relevant financial or non-financial competing interests.<br><br>This statement is required for submission and **will appear in the published article** if the submission is accepted. Please make sure it is accurate and that any funding sources listed in your Funding Information later in the submission form are also	The authors have declared that no competing interests exist.

Powered by Editorial Manager® and ProduXion Manager® from Aries Systems Corporation

declared in your Financial Disclosure statement.<br><br>View published research articles from *PLOS ONE* for specific examples.<br><br>**NO authors have competing interests**<br><br>Enter: *The authors have declared that no competing interests exist.*<br><br>**Authors with competing interests**<br><br>Enter competing interest details beginning with this statement:<br><br>*I have read the journal's policy and the authors of this manuscript have the following competing interests: [insert competing interests here]*<br><br>* typeset	
**Ethics Statement**<br><br>Enter an ethics statement for this submission. This statement is required if the study involved:<br><br>• Human participants<br>• Human specimens or tissue<br>• Vertebrate animals or cephalopods<br>• Vertebrate embryos or tissues<br>• Field research<br><br>Write "N/A" if the submission does not require an ethics statement.<br><br>General guidance is provided below. Consult the submission guidelines for detailed instructions. **Make sure that all information entered here is included in the Methods section of the manuscript.**	The secondary data used in this study was gathered by Bangladesh's NIPORT and MEASURE DHS. The ethical guidelines of the national research committee, the 1964 Helsinki Declaration and its later amendments, or equivalent ethical standards were followed during all procedures carried out in this study that involved human subjects. According to local law and institutional regulations, an ethical evaluation and permission were not necessary for the study on human participants because the data is publicly available on the website.

Powered by Editorial Manager® and ProduXion Manager® from Aries Systems Corporation

**Format for specific study types**<br><br>**Human Subject Research (involving human participants and/or tissue)**<br>• Give the name of the institutional review board or ethics committee that approved the study<br>• Include the approval number and/or a statement indicating approval of this research<br>• Indicate the form of consent obtained (written/oral) or the reason that consent was not obtained (e.g. the data were analyzed anonymously)<br><br>**Animal Research (involving vertebrate animals, embryos or tissues)**<br>• Provide the name of the Institutional Animal Care and Use Committee (IACUC) or other relevant ethics board that reviewed the study protocol, and indicate whether they approved this research or granted a formal waiver of ethical approval<br>• Include an approval number if one was obtained<br>• If the study involved *non-human primates*, add *additional details* about animal welfare and steps taken to ameliorate suffering<br>• If anesthesia, euthanasia, or any kind of animal sacrifice is part of the study, include briefly which substances and/or methods were applied<br><br>**Field Research**<br><br>Include the following details if this study involves the collection of plant, animal, or other materials from a natural setting:<br>• Field permit number<br>• Name of the institution or relevant body that granted permission	
**Data Availability**<br><br>Authors are required to make all data underlying the findings described fully available, without restriction, and from the time of publication. PLOS allows rare exceptions to address legal and ethical concerns. See the PLOS Data Policy and FAQ for detailed information.	Yes - all data are fully available without restriction

A Data Availability Statement describing where the data can be found is required at submission. Your answers to this question constitute the Data Availability Statement and **will be published in the article**, if accepted.<br><br>**Important:** Stating 'data available on request from the author' is not sufficient. If your data are only available upon request, select 'No' for the first question and explain your exceptional situation in the text box.<br><br>Do the authors confirm that all data underlying the findings described in their manuscript are fully available without restriction?	
**Describe where the data may be found in full sentences. If you are copying our sample text, replace any instances of XXX with the appropriate details.**<br><br>• If the data are **held or will be held in a public repository**, include URLs, accession numbers or DOIs. If this information will only be available after acceptance, indicate this by ticking the box below. For example: *All XXX files are available from the XXX database (accession number(s) XXX, XXX.*).<br>• If the data are all contained **within the manuscript and/or Supporting Information files**, enter the following: *All relevant data are within the manuscript and its Supporting Information files.*<br>• If neither of these applies but you are able to provide **details of access elsewhere**, with or without limitations, please do so. For example:<br><br>*Data cannot be shared publicly because of [XXX]. Data are available from the XXX Institutional Data Access / Ethics Committee (contact via XXX) for researchers who meet the criteria for access to confidential data.*<br><br>*The data underlying the results presented in the study are available from (include the name of the third party*	Data are available in a public, open access repository. The BDHS 2017–2018 data are publicly accessible on request from the DHS website at https://dhsprogram.com/data/available-datasets.cfm.

and contact information or URL).<br>• This text is appropriate if the data are owned by a third party and authors do not have permission to share the data.<br><br>* typeset	
Additional data availability information:	

Powered by Editorial Manager® and ProduXion Manager® from Aries Systems Corporation

Manuscript Click here to access/download;Manuscript;Main Manuscript.docx

Rural and Urban Differences in the Prevalence of Type-2 Diabetes and the Associated Risk Factors: Analysis of a Population-Based Nation-wide Cross-sectional Survey in Bangladesh

Author order	Author Name (First name, Middle name initial followed by Family name)	Affiliation (Department, Institution, City, State, Country)
1.	Ashis Talukder	Assistant Professor, Statistics Discipline, Science Engineering and Technology School, Khulna University, Khulna-9208, Bangladesh.<br>&<br>PhD Researcher, National Centre for Epidemiology and Population Health, Australian National University<br>Email:<br>ashistalukder3168@ku.ac.bd;<br>ashis.talukder@anu.edu.au<br>ORCID: https://orcid.org/0000-0002-2205-0696
2.	Sabiha Shirin Sara	Statistics Discipline, Science Engineering and Technology School, Khulna University, Khulna-9208, Bangladesh.<br>Email: shirinsara11@gmail.com
3.	Md. Tanvir Hossain	Associate Professor, Sociology Discipline, Social Science School, Khulna University, Khulna-9208, Bangladesh.<br>Email: tanvirku05@soc.ku.ac.bd<br>ORCiD: https://orcid.org/0000-0003-0315-6900
4.	Chuton Deb Nath	Assistant Professor, Mass Communication and Journalism Discipline, Social Science School, Khulna University, Khulna-9208, Bangladesh.<br>Email: chutondebnath@mcj.ku.ac.bd
5.	Riaz Rahman	Statistics Discipline, Science Engineering and Technology School, Khulna University, Khulna-9208, Bangladesh.<br>Email: rizurahman758@gmail.com<br>Orcid: https://orcid.org/0000-0001-6683-5906
6.	Sadiq Hussain	Examination Branch<br>Dibrugarh University, India<br>Email: sadiq@dibru.ac.in
7.	Md. Nazmul Huda[1,2]	[1]ARCED Foundation, 13/1, Pallabi, Mirpur-12, Dhaka 1216, Bangladesh<br>[2]Translational Health Research Institute, Western Sydney University, Campbell town, NSW 2560, Australia<br>Email: nazmul.Huda@westernsydney.edu.au

Correspondence:

1.	Ashis Talukder	Assistant Professor, Statistics Discipline, Science Engineering and Technology School, Khulna University, Khulna-9208, Bangladesh.<br>Email: ashistalukder3168@ku.ac.bd<br>ORCID: https://orcid.org/0000-0002-2205-0696

Abstract

Objective: To estimate the prevalence of T2D in both urban and rural settings and identify the risk factors specific to each location.

Method: We explore the Bangladesh Demographic and Health Survey 2017-18 data sourcing from DHS website. A stratified two-stage sample was used for this survey where 7658 women and 7048 men aged 18 and older had found their blood glucose levels measured. Chi-square test and ordinal logistic regression were used to find the association between selected variables of both urban and rural settings with diabetes and prediabetes.

Results: The prevalence of diabetes is 10.8% and 7.4%, and pre-diabetes is 31.4% and 27% in urban and rural areas, respectively. The study identifies significant influences on diabetes of 55-64 age group in both urban and rural settings (Urban: AOR= 1.882, CI [1.462, 2.421]; Rural: AOR=1.874, CI [1.545,2.273]). The odds of diabetes were lower among highly educated participants, and higher among rich and overweight participants in both areas. In rural areas, the odds of diabetes is higher among caffeinators and physically inactive participants, whilst those show insignificant influence in urban areas. Moreover, the participants from urban areas show a considerable association between diabetes and hypertension.

Conclusion: Our findings urge the importance of location specific imminent preventive measures for T2D and prediabetes considering the concentration of the socio-demographic risk factors in both urban and rural contexts which essentially demand a sound public health policy to prevent the looming threat of public health sector.

Key Words: Type 2 Diabetes, Urban, Rural, Risk factors, Cross-sectional Survey, Bangladesh

Introduction

Diabetes is a chronic condition, a leading contributor to heart disease, stroke, blindness, neuropathy, kidney disease, and amputations. Diabetes typically presents in three ways:, Type 1 diabetes (T1D) is an autoimmune disease where the immune system assaults and destroys insulin-producing beta cells in the pancreas, whereas gestational diabetes (GDM) occurs during pregnancy as a result of hormonal changes that decrease insulin sensitivity. In addition, type 2 diabetes (T2D), also known as non-insulin-dependent diabetes, is brought on by the body's ineffective usage of the hormone insulin [1, 2]. Because of the impaired insulin ejection by pancreatic cells, which typically occurs against pre-existing insulin resistance in skeletal muscle, the liver, and adipose tissue, T2D is the category that affects most persons with diabetes [3]. Globally, the estimated diabetes prevalence is 10.5% in 2021 and is anticipated to increase to 12.2%

by 2045 [4]. Specifically, diabetes is affecting almost one in ten persons in Bangladesh [5], a public health concern.

Globally, the prevalence of T2D has been growing rapidly, especially in regions like Asia, the Middle East, and North Africa [6]. It is presumable that the burden of death and disability is significantly impacted by T2D consequences. Additionally, diabetes was listed as the tenth main factor affecting life expectancy [7]. T2D elevates the risk of dementia [8], cancer by around a two-fold margin [9, 10], and cardiovascular disease [11]. Depression and platelet dysfunction have also been linked to T2D [12, 13].

Due to the food habits and lifestyle of the region, T2D is spreading alarmingly throughout South Asia [14]. Bangladesh, one of the most densely populated countries, has the second-highest percentage of diabetic adults (6.31%) in the region [15]. In Bangladesh, 8.4 million individuals have diabetes in 2019; by 2045, that number is projected to be doubled to 15.0 million [16]. Pre-diabetes is predicted to affect 3.8 million individuals in Bangladesh in 2019 [16]. Pre-diabetes has been on the rise, according to most of the research done in Bangladesh [5], with low treatment and control rates [15]. Various unfavorable living situations, including crowded living conditions in slums specially in urban and associated stress, may contribute to increasing diabetes mellitus[17, 18]. Therefore, examining the variations in T2D prevalence and associated risk factors between an urban and rural population is intriguing.

In Bangladesh, there is a growing body of studies examining T2D and its risk factors. For example, several studies demonstrated education level, hypertension, financial status, physical activity, abdominal obesity, social class, family history, waist-hip ratio, and urbanization as some of the risk factors linked to diabetes in Bangladesh [19, 20, 21, 22]. Furthermore, other studies identified gender [10, 11, 8], high blood pressure [17], older age [23], way of life [13, 24], BMI [25], and ethnicity [26] as the risk factors for diabetes. Evidence also indicates an annual increase of diabetes rates in both urban (0.06%) and rural (0.05%) settings [27]. However, there are limited studies of diabetes prevalence and its associated risk factors using nationally representative surveys in urban and rural settings separately. Therefore, our study aims to measure the prevalence of T2D in both urban and rural settings and identify the risk factors specific to each location.

Methods

Data Source

Secondary data from the BDHS (Bangladesh Demographic and Health Survey) in 2017–18 were used in this study. The 2017–18 BDHS data were administered by the National Institute for Population Research and Training (NIPORT), and they are accessible on the DHS programme website [28]. The existing variable "Type of place of residence" served as the basis for our study's division of the dataset into urban and rural areas.

Sampling design

For both contexts, a stratified two-stage home sample was used. The Bangladesh Bureau of Statistics (BBS) took the sample in the initial stage using probability proportionate to size based on an area. In order to create a framework for sampling, a comprehensive household census was carried out in all chosen enumeration areas (EAs) in the second stage. 20,250 homes made up the 2017–18 sample, and 19,457 of those successfully completed the interview. The study was finished in 672 clusters after the removal of

three due to erosion caused by floodwaters. This study found that 87% of women and 80% of men who were eligible to have their blood glucose levels measured had their blood glucose checked, out of 7658 women and 7048 men who were 18 years of age or older.

Selection of sample

Figure 1 shows the procedures of the selection of the samples. Among the total participants, 12300 were found as eligible for diabetes measurement. Then, we split the eligible participants into two portions: Urban and Rural based on the type of place of residence and finally conducted our analysis.

Dependent variable

The responders were instructed to fast for at least eight hours before to the test in order to test their fasting plasma blood glucose levels. The acquired value was converted to fasting plasma glucose equivalent values using the HemoCue Gucose 201 Dm system [29]. Then, in our study, we classified the fasting plasma glucose values into three categories in accordance with the recommendations of the World Health Organisation (WHO), and we called the dependent variable "Diabetes Status" in order to determine the presence of diabetes. Here, fasting blood glucose levels between 70 mg/dL (3.9 mmol/L) and 100 mg/dL (5.6 mmol/L) were regarded as normal, while those between 100 and 125 mg/dL (5.6 to 6.9 mmol/L) were classified as prediabetes and those above 126 mg/dL (7 mmol/L) as diabetic [20, 30].

Explanatory variables

Gender, age, education level, wealth index, smoking habit, consumption of caffeinated beverages, physical activity, hypertension, and BMI were explanatory variables. The selection of these factors and their categorisation was made after a thorough assessment of earlier studies from Bangladesh and other countries. Age is a numerical variable that has been broken down into the following four groups: youth (15–24 years) [31], prime working age (25–54 years), mature working age (55–64 years), and elder (65 years and above) [32]. Using an existing occupation type variable as a starting point, we developed the categorical variable "physical activity." Physically inactive respondents included those with desk-based occupations like doctors, attorneys, businesspeople, and the unemployed. Respondents who primarily engaged in physical labour, like farmers, fishermen, and manufacturing employees, were categorised as being physically active [33]. Another variable BMI was calculated by the DHS program. They collected data on heights and weights. Heights were measured standing up and weights were measured using SECA scales with a digital display. Then, bmi was calculated as weight in kilograms divided by the square of height in meters. To make our study more meaningful, we then categorized BMI into three categories. BMI with 18.5 kg/m^2 was considered underweight, BMI from 18.5 to 22.9 was normal, and BMI ≥ 23 was overweight [34]. Again, the measurement of blood pressure was taken at three different times, at about 10 min intervals, by trained health technicians. Then, the average of the measurements was granted as the final measurement for BP. The respondents with systolic blood pressure (SBP) < 140 and diastolic blood pressure (DBP) < 90 were considered to have no hypertension. If one violates the above rules, he/she has hypertension [35].

Statistical tools

The study requires model adjustment to detect risk factors for pre-diabetes and diabetes and raise awareness about lifestyle. In univariate analysis, a frequency distribution table was used to describe data.

Chi-square test was used to find the association between variables. In multivariable analysis, an ordinal logistic regression model was fitted. The data analysis was conducted using R (version 4.1.0). A 95% confidence interval was used to interpret the regression analysis with a significance level set at $p < 0.05$.

Funding source

This study receives no fund from any source. The dataset was fully accessible to all the authors and they were fully agree on the final submission for publication.

Results

Table 1 describes the basic characteristics of the participants for both urban and rural areas. Male participants (53.2%) were higher in urban areas, while female participants (55.3%) were higher in rural areas. Regarding education level, around 30% of participants in urban areas had secondary education, and for rural participants, more than 30% participants had primary education. Regarding caffeinated drinks, the urbanites had a higher percentage (9.6%), whereas smoking was higher among the rural people (17%). Comparatively, the percentage of hypertension (25.1%) and overweight (50.2%) was higher among urban participants, whereas rural participants (43%) were more physically inactive. Among the urban participants, 43.7% were richest but in rural areas, the percentage of richest participants was 9.8%. The percentages of diabetes were higher among urban participants (10.8%) than the rural participants (7.4%).

Table 2 further shows the association of socio-demographic variables with diabetes. Age showed a significant association with diabetes for both urban $(\chi^2 = 93.232, p < 0.01)$ and rural $(\chi^2 = 93.232, p < 0.01)$ areas. Participants of working age had the highest percentages of having diabetes compared to the other age groups for both urban and rural areas. The caffeinated drinks also showed a significant association with diabetes in both urban $(\chi^2 = 7.988, p < 0.05)$ and rural $(\chi^2 = 13.478, p < 0.01)$ areas. The percentage of diabetes is lower for the participants who took a caffeinated drink for both urban and rural areas with 13% and 7%, respectively. In both urban $(\chi^2 = 270.2, p < 0.01)$ and rural $(\chi^2 = 110.80, p < 0.01)$ areas, division was significantly associated with diabetes. For urban areas, Dhaka showed the highest percentages for both diabetes (30%) and pre-diabetes (31%); in contrast, for rural areas, Chittagong had the highest percentages for both diabetes (17%) and pre-diabetes (14%). Another risk factor associated with diabetes is hypertension that showed a statistically significant association $(\chi^2 = 80.643, p < 0.01)$ and $(\chi^2 = 76.271, p < 0.01)$ for both urban and rural areas, respectively. The prevalence of hypertension among diabetes participants is higher among urban participants (42%) than that of rural participants (39%). Moreover, body mass index, physical activity, and wealth status also showed a significant association with diabetes status in urban and rural areas. The prevalence of overweight is relatively higher among urban diabetes participants (65%) than rural diabetes participants (53%). Moreover, 83% of urban diabetes participants were physically inactive, but the prevalence is lower among rural participants (67%). Regarding the wealth status, the prevalence of diabetes was higher among the richest urban participants (59%) than the richest participants in rural areas (23%). The prevalence of pre-diabetes is higher among the poorest rural participants (24%) than the poorest urban participants (5%).

Table 3 shows the ordinal logistic regression analysis predicting diabetes in urban and rural areas. Age showed a significant association with diabetes in urban areas, where participants at primary working age, working age and older age were 1.35[illegible]882, and 1.645 times more likely to have diabetes compared to the young, aged participants. In case of rural areas, participants at primary working age, working age, and

older age were 1.561, 1.874, and 1.523 times more likely to develop diabetes than the young participants. Higher education levels showed a significant association with diabetes in urban and rural areas, where participants with higher education were 0.673 and 0.805 times less likely to develop diabetes, respectively, compared to the participants did not attend any school. Body mass index also showed a significant association with risk of developing diabetes. In fact, the overweight participants were 1.399 and 1.318 times more likely to develop diabetes compared to the underweight participants in urban and rural areas, respectively. Moreover, wealth status also showed a significant association with diabetes. Richer (2.647 and 1.397) and richest (4.165 and 2.152) participants showed a significant association with diabetes in both urban and rural areas, respectively. Moreover, middle participants were also 1.761 times more likely to develop diabetes compared to the poorest participants in urban areas, but it was not significant for rural areas. Physical activity showed a significant association for rural areas but showed an insignificant association for urban areas. It specified that physically inactive participants were 1.294 times more likely to develop diabetes compared to the physically active participants. Moreover, hypertension also showed a significant association with diabetes in urban areas but showed an insignificant association in rural areas. Participants who had hypertension were 1.256 times more likely to develop diabetes compared to the participants who did not have hypertension. The caffeinated drink also showed a significant association with diabetes in rural areas. Participants who took caffeinated drinks were 1.243 times more likely to develop diabetes than the participants who did not take any caffeinated drinks in rural areas.

Discussion

In both urban and rural locations, there was a significant correlation between participants' ages and diabetes. Results show that participants in both urban and rural settings who were older and of working age had a higher chance of developing diabetes than those who were younger. This may occur as a result of the interactions between rising insulin resistance, an unhealthy lifestyle, and their inability to satisfy daily physical activity recommendations. [36, 37].

According to previous study, those with low levels of education are more prone to acquire T2D [38]. As literacy may lessen the considerable difficulties and problems associated with this condition, our findings show that those with a high degree of education were less likely to have diabetes than participants with no education, both in urban and rural locations [39].

The body mass index (BMI) is a significant risk factor for developing diabetes. In both urban and rural patients in our study, there was a strong correlation between BMI and diabetes. Our results showed that participants with overweight were more likely to have diabetes in both urban and rural areas than participants with underweight. According to a US study [39], BMI plays a significant role in predicting the likelihood of acquiring diabetes, and a subsequent study from Bangladesh supports this conclusion [40].

Through the inverse connection shown in other studies [41], we found that caffeine use increases the probability of getting T2D in rural areas. As caffeine is unfamiliar in rural areas, the consumption of caffeine may disrupt the blood sugar regulation and this contributes the development of insulin resistance. However, caffeine-containing beverages had no significant association with diabetes in urban regions. This is possible due to urban individuals’ access to and preference for caffeinated beverages on regular basis and for this, over time, one’s body may develop tolerance to it.

Diabetes was also found to be highly associated with wealth status. In both urban and rural locations, diabetes was found to be substantially related with participants who were both richer and the richest. This happens because of the habitant of inactivity and excessive consumption of high-calorie foods among richer and richest participants. However, only in urban settings did it come to light that participants with middle wealth status had a strong association with diabetes. The risk of having diabetes may be influenced by the luxury and readily accessible or available life of urban middle-class individuals compared to rural people. [44,42]

In rural areas, it is apparent that physical exercise showed a substantial connection with diabetes. According to a study, regular exercise may lower the incidence of T2D [43]. Our findings concur with the earlier investigation. Compared to the physically active participants, the rural physically inactive participants were more likely to develop diabetes. The fundamental cause of this is that physically active people keep their bodies free of cholesterol. Moreover, physical activities may low the blood glucose level and boost ones body to sensitivity to insulin, countering insulin resistance [44].

In our investigation, systolic and diastolic blood pressure was utilized to assess whether it influences the risk of diabetes. Urban participants exhibit a strong association between hypertension and diabetes, whereas individuals in rural regions exhibit an insignificant association. Another investigation does not show any connection between diabetes and hypertension. [45]

Strength and limitations

Our study elucidates the compared scenario of diabetes between urban and rural areas including associated risk factors with prevalence and estimated values. This study includes the latest Demographic and Health survey data to estimate diabetes in Bangladesh. That disclose the strength of our study. Besides this strength, the study also has some limitations. Large number of the missing values and the constricted risk factors indicate one of the limitations of this study. Moreover, the information about the other types of diabetes are also unavailable. That can also be considered as another limitation of our study.

Generalisability

Our findings visualize the considerable distinct prevalence of T2D and prediabetes in urban and rural areas focusing socio-demographic associated risk factors like age, education level, wealth status, physical activity, consumption of caffeinated beverage, hypertension and BMI etc. The prevalence of T2D and prediabetes in urban setting 10.8% and 31.4% whereas in rural 7.4% and 27%, respectively which clearly indicates the rural setting have a lower prevalence of diabetes and prediabetes than urban setting. From our study, it is also apparent that the residents of urban environment are twice more likely to develop diabetes compared to the rural environment.

Our study outcomes impulse for the coordinating efforts of the epistemo[illegible]sts, public health researchers, policy makers, health practitioners, NGOs and other relevant stakeholders to formulate a sound public health policy considering the different prevalence in the urban and rural contexts. Preventive measures like effective health education program, awareness building campaign and most importantly a healthy public policy are must to curb the alarming rising rate of T2D and prediabetes. Polices should highlight the exorbitant associated factors of T2D among urban residents and rural residents separately and advocate the possible means of preventive interventions as both settings demand unique actions.

Conclusion

Our study substantially conclude that individuals live in metropolitan setting have a higher prevalence of diabetes than those from rural setting and major individuals in both urban (31.4%) and rural (27%) settings are identified as prediabetes which poses an imminent danger for the public health sector. Our findings echo with other studies that the percentage of diabetic patients will increase if an awareness building-campaign and some other preventive measures are not taken, considering both rural and urban situations to prevent the alarming rate. This study extends our knowledge to compare the prevalence of Type 2 Diabetes in both rural and urban settings. Epidemiologists, health policy makers and the researchers need to work together to develop a comprehensive programs to deal with the looming threat of T2D putting paramount importance on increasing public awareness.

Funding: No fund has been received.

Conflict of interest: The authors have no conflicts of interest to declare.

References

[1] F. Wu and P. Liang, "Application of Metabolomics in Various Types of Diabetes," *Diabetes Metab Syndr Obes*, vol. 15, pp. 2051–2059, Jul. 2022, doi: 10.2147/DMSO.S370158.

[2] A. B. Olokoba, O. A. Obateru, and L. B. Olokoba, "Type 2 Diabetes Mellitus: A Review of Current Trends," *Oman Med J*, vol. 27, no. 4, pp. 269–273, Jul. 2012, doi: 10.5001/omj.2012.68.

[3] R. A. DeFronzo *et al.*, "Type 2 diabetes mellitus," *Nat Rev Dis Primers*, vol. 1, no. 1, Art. no. 1, Jul. 2015, doi: 10.1038/nrdp.2015.19.

[4] H. Sun *et al.*, "IDF Diabetes Atlas: Global, regional and country-level diabetes prevalence estimates for 2021 and projections for 2045," *Diabetes Research and Clinical Practice*, vol. 183, p. 109119, Jan. 2022, doi: 10.1016/j.diabres.2021.109119.

[5] S. Akter, M. M. Rahman, S. K. Abe, and P. Sultana, "Prevalence of diabetes and prediabetes and their risk factors among Bangladeshi adults: a nationwide survey," *Bull World Health Organ*, vol. 92, pp. 204-213A, Jan. 2014, doi: 10.2471/BLT.13.128371.

[6] M. G. Tinajero and V. S. Malik, "An Update on the Epidemiology of Type 2 Diabetes: A Global Perspective," *Endocrinology and Metabolism Clinics of North America*, vol. 50, no. 3, pp. 337–355, Sep. 2021, doi: 10.1016/j.ecl.2021.05.013.

[7] Y. Zheng, S. H. Ley, and F. B. Hu, "Global aetiology and epidemiology of type 2 diabetes mellitus and its complications," *Nat Rev Endocrinol*, vol. 14, no. 2, Art. no. 2, Feb. 2018, doi: 10.1038/nrendo.2017.151.

[8] M. Barbagallo and L. J. Dominguez, "Type 2 diabetes mellitus and Alzheimer's disease," *World journal of diabetes*, vol. 5, no. 6, p. 889, 2014.

[9] C. Nwaneri, H. Cooper, and D. Bowen-Jones, "Mortality in type 2 diabetes mellitus: magnitude of the evidence from a systematic review and meta-analysis," *The British Journal of Diabetes & Vascular Disease*, vol. 13, no. 4, pp. 192–207, 2013.

[10] A. López-Suárez, "Burden of cancer attributable to obesity, type 2 diabetes and associated risk factors," *Metabolism*, vol. 92, pp. 136–146, 2019.

[11] J. Barbieri *et al.*, "Anemia en pacientes con diabetes mellitus tipo 2," *Hindawi. doi: https://doi. org/10.1155/2015/354737*, 2015.

[12] A. I. Vinik, T. Erbas, T. S. Park, R. Nolan, and G. L. Pittenger, "Platelet dysfunction in type 2 diabetes," *Diabetes care*, vol. 24, no. 8, pp. 1476–1485, 2001.

[13] J. F. Téllez-Zenteno and M. H. Cardiel, "Risk factors associated with depression in patients with type 2 diabetes mellitus," *Archives of medical research*, vol. 33, no. 1, pp. 53–60, 2002.

[14] A. Afroz *et al.*, "The impact of diabetes on the productivity and economy of Bangladesh," *BMJ Global Health*, vol. 5, no. 6, p. e002420, 2020.

[15] M. S. Rahman *et al.*, “Awareness, treatment, and control of diabetes in Bangladesh: a nationwide population-based study,” *PloS one*, vol. 10, no. 2, p. e0118365, 2015.
[16] N. H. Cho *et al.*, “IDF Diabetes Atlas: Global estimates of diabetes prevalence for 2017 and projections for 2045,” *Diabetes research and clinical practice*, vol. 138, pp. 271–281, 2018.
[17] A. Talukder and M. Z. Hossain, “Prevalence of diabetes mellitus and its associated factors in Bangladesh: application of two-level logistic regression model,” *Scientific Reports*, vol. 10, no. 1, p. 10237, 2020.
[18] S. J. Han and E. J. Boyko, “The evidence for an obesity paradox in type 2 diabetes mellitus,” *Diabetes & Metabolism Journal*, vol. 42, no. 3, pp. 179–187, 2018.
[19] D. M. Nathan and D. R. Group, “The diabetes control and complications trial/epidemiology of diabetes interventions and complications study at 30 years: overview,” *Diabetes care*, vol. 37, no. 1, pp. 9–16, 2014.
[20] D. S. Alam *et al.*, “Overweight and abdominal obesity as determinants of undiagnosed diabetes and pre-diabetes in Bangladesh,” *BMC obesity*, vol. 3, pp. 1–12, 2016.
[21] T. Sal-Sabil, A. Islam, and S. S. Islam, “Risk factors for type 2 diabetes in Bangladesh: a systematic review,” *Journal of Diabetology*, vol. 7, no. 2, p. 5, 2016.
[22] M. A. Islam, M. M. R. Khan, P. M. Basak, M. M. Khanam, and A. A. Masum, “Prevalence and risk factors of Type 2 diabetes in an urbanizing rural community of Bangladesh,” *TAJ: Journal of Teachers Association*, vol. 29, no. 2, pp. 26–30, 2016.
[23] E. F. F. Silva, C. M. M. Ferreira, and L. de Pinho, “Risk factors and complications in type 2 diabetes outpatients,” *Revista da Associação Médica Brasileira*, vol. 63, pp. 621–627, 2017.
[24] M. Muilwijk, R. Bolijn, H. Galenkamp, K. Stronks, E. M. van Charante, and I. G. van Valkengoed, “The association between gender-related characteristics and type 2 diabetes risk in a multi-ethnic population: The HELIUS study,” *Nutrition, Metabolism and Cardiovascular Diseases*, vol. 32, no. 1, pp. 142–150, 2022.
[25] Z. Tian *et al.*, “Gender-specific associations of body mass index and waist circumference with type 2 diabetes mellitus in Chinese rural adults: The Henan Rural Cohort Study,” *Journal of Diabetes and its Complications*, vol. 32, no. 9, pp. 824–829, 2018.
[26] B. S. Nayak *et al.*, “The association of age, gender, ethnicity, family history, obesity and hypertension with type 2 diabetes mellitus in Trinidad,” *Diabetes & Metabolic Syndrome: Clinical Research & Reviews*, vol. 8, no. 2, pp. 91–95, 2014.
[27] T. Biswas, A. Islam, L. B. Rawal, and S. M. S. Islam, “Increasing prevalence of diabetes in Bangladesh: a scoping review,” *Public health*, vol. 138, pp. 4–11, 2016.
[28] “The DHS Program - Available Datasets.” https://dhsprogram.com/data/available-datasets.cfm (accessed Apr. 25, 2023).
[29] N. I. of P. R. and T.- Niport, M. of H. and F. Welfare, and ICF, “Bangladesh Demographic and Health Survey 2017-18,” Oct. 2020, Accessed: Dec. 14, 2022. [Online]. Available: https://dhsprogram.com/publications/publication-FR344-DHS-Final-Reports.cfm
[30] “Mean fasting blood glucose.” https://www.who.int/data/gho/indicator-metadata-registry/imr-details/2380 (accessed Apr. 25, 2023).
[31] “Adolescent health.” https://www.who.int/southeastasia/health-topics/adolescent-health (accessed Aug. 11, 2022).
[32] “Bangladesh Age structure - Demographics.” https://www.indexmundi.com/bangladesh/age_structure.html (accessed Aug. 11, 2022).
[33] K. E. Dalene *et al.*, “Occupational physical activity and longevity in working men and women in Norway: a prospective cohort study,” *Lancet Public Health*, vol. 6, no. 6, pp. e386–e395, Jun. 2021, doi: 10.1016/S2468-2667(21)00032-3.
[34] J. U. Lim *et al.*, “Comparison of World Health Organization and Asia-Pacific body mass index classifications in COPD patients,” *Int J Chron Obstruct Pulmon Dis*, vol. 12, pp. 2465–2475, 2017, doi: 10.2147/COPD.S141295.
[35] G. M. Al Kibria, V. Burrowes, A. Choudhury, A. Sharmeen, and K. Swasey, “Sex differences in prevalence and associated factors of prehypertension and hypertension among Bangladeshi adults,” *International Journal of Cardiology Hypertension*, vol. 1, p. 100006, May 2019, doi: 10.1016/j.ijchy.2019.100006.

[36] M. B. Weber, H. Ranjani, G. C. Meyers, V. Mohan, and K. V. Narayan, "A model of translational research for diabetes prevention in low and middle-income countries: The Diabetes Community Lifestyle Improvement Program (D-CLIP) trial," *Primary Care Diabetes*, vol. 6, no. 1, pp. 3–9, 2012.
[37] S. Bellary, I. Kyrou, J. E. Brown, and C. J. Bailey, "Type 2 diabetes mellitus in older adults: clinical considerations and management," *Nat Rev Endocrinol*, vol. 17, no. 9, Art. no. 9, Sep. 2021, doi: 10.1038/s41574-021-00512-2.
[38] C. Sacerdote *et al.*, "Lower educational level is a predictor of incident type 2 diabetes in European countries: the EPIC-InterAct study," *International journal of epidemiology*, vol. 41, no. 4, pp. 1162–1173, 2012.
[39] M. L. Ganz, N. Wintfeld, Q. Li, V. Alas, J. Langer, and M. Hammer, "The association of body mass index with the risk of type 2 diabetes: a case–control study nested in an electronic health records system in the United States," *Diabetology & metabolic syndrome*, vol. 6, no. 1, pp. 1–8, 2014.
[40] M. M. Rahman, S. Akter, J. Jung, M. S. Rahman, and P. Sultana, "Trend, projection, and appropriate body mass index cut-off point for diabetes and hypertension in Bangladesh," *Diabetes research and clinical practice*, vol. 126, pp. 43–53, 2017.
[41] S. N. Bhupathiraju *et al.*, "Caffeinated and caffeine-free beverages and risk of type 2 diabetes," *The American journal of clinical nutrition*, vol. 97, no. 1, pp. 155–166, 2013.
[42] N. Saquib *et al.*, "High prevalence of type 2 diabetes among the urban middle class in Bangladesh," *BMC Public Health*, vol. 13, p. 1032, Oct. 2013, doi: 10.1186/1471-2458-13-1032.
[43] H. Hamasaki, "Daily physical activity and type 2 diabetes: A review," *World journal of diabetes*, vol. 7, no. 12, p. 243, 2016.
[44] P. Wiklund, "The role of physical activity and exercise in obesity and weight management: Time for critical appraisal," *Journal of Sport and Health Science*, vol. 5, no. 2, pp. 151–154, Jun. 2016, doi: 10.1016/j.jshs.2016.04.001.
[45] D. Sun *et al.*, "Type 2 diabetes and hypertension: a study on bidirectional causality," *Circulation research*, vol. 124, no. 6, pp. 930–937, 2019.

Tables

Table 1. Showing [illegible] frequency distribution of the selected variables

Covariates	Category	Urban		Rural	
		Frequency	Percentages	Frequency	Percentages

**Gender**	**Male**	2934	53.2	4114	44.7
	**Female**	2577	46.8	5081	55.3
**Age**	**Youth**	1176	21.3	1828	19.9
	**Prime working**	3327	60.4	5228	56.9
	**Mature working**	556	10.1	1105	12.0
	**Older**	452	8.2	1034	11.2
**Education level**	**No education**	1043	18.9	2668	29.0
	**Primary**	1467	26.6	2861	31.1
	**Secondary**	1636	29.7	2557	27.8
	**Higher**	1361	24.7	1099	12.0
**Caffeinated drink**	**No**	4317	90.4	7812	93.5
	**Yes**	461	9.6	541	6.5
**Smoking Status**	**No**	4137	86.6	6931	83.0
	**Yes**	641	13.4	1421	17.0
**Division**	**Barisal**	2966	9.2	6582	11.4
	**Chittagong**	4923	15.2	8413	14.6
	**Dhaka**	7409	22.9	5387	9.4
	**Khulna**	3949	12.2	7016	12.2
	**Mymensingh**	2541	7.9	7564	13.1
	**Rajshahi**	3497	10.8	7276	12.6
	**Rangpur**	3189	9.9	7526	13.1
	**Sylhet**	3817	11.8	7764	13.5
**Hypertension**	**No**	3577	74.9	6331	75.8
	**Yes**	1199	25.1	2024	24.2
**BMI**	**Underweight**	599	12.6	1609	19.5
	**Normal**	1762	37.1	3658	44.3
	**Overweight**	2382	50.2	2990	36.2
**Physically Active**	**Yes**	1359	75.1	3919	57.0
	**No**	4089	24.9	5203	43.0
**Wealth status**	**Poorest**	2951	9.1	15110	26.3
	**Poorer**	2698	8.4	14601	25.4
	**Middle**	4359	13.5	12688	22.1
	**Richer**	8187	25.4	9517	16.5
	**Richest**	14096	43.7	5612	9.8
**Diabetes Status**	**Normal**	2539	57.8	5182	65.5
	**Pre-diabetes**	1381	31.4	2138	27.0
	**Diabetes**	473	10.8	587	7.4

Table 2. Bivariate analysis of the selected variables for urban and rural areas

Covariates	Urban				Rural			
	Blood Glucose				Blood glucose			
	Normal	Pre-diabetes n (%)	Diabetes n (%)	Chi-square (p-value)	Normal	Pre-diabetes n (%)	Diabetes n (%)	Chi-square (p-value)
**Gender**								
Male	1152(45)	597(43)	200(42)	2.5953 (0.2732)	2198(42)	886(41)	265(45)	2.6134 (0.2707)
Female	1387(55)	784(57)	273(58)		2983(58)	1252(59)	322(55)	
**Age**								
Young	610(24)	272(20)	37(8)	**89.398 (<0.001)**	1129(22)	356(17)	50(9)	**93.232 (<0.001)**
Working	1523(60)	847(61)	300(63)		2910(56)	1279(60)	344(59)	
Primary working	210(8)	146(11)	74(16)		582(11)	273(13)	109(19)	
Older	196(8)	116(8)	62(13)		560(11)	230(11)	84(14)	
**Education level**								
No education	498(20)	268(19)	87(18)	5.0437 (0.5382)	1513(29)	599(28)	165(28)	3.8792 (0.693)
Primary	697(27)	390(28)	124(26)		1619(31)	678(32)	197(34)	
Secondary	782(31)	396(29)	159(34)		1430(28)	610(29)	166(28)	
Higher	562(22)	327(24)	103(22)		619(12)	251(12)	59(10)	
**Caffeinated drink**								
No	2323(92)	1258(91)	412(87)	**7.9886 (0.018)**	4893(95)	1974(92)	545(93)	**13.478 (0.001)**
Yes	215(8)	123(9)	59(13)		282(5)	163(8)	42(7)	
**Smoking status**								
Yes	2207(87)	1215(88)	405(86)	1.5009 (0.4721)	4326(84)	1788(84)	478(82)	1.6516 (0.4379)
No	331(13)	166(12)	66(14)		849(16)	349(16)	108(18)	
**Division**								
Barisal	237(9)	140(10)	46(10)	**270.2 (<0.01)**	537(10)	256(12)	65(11)	**110.8 (<0.01)**
Chittagong	368(14)	198(14)	66(14)		633(12)	302(14)	100(17)	
Dhaka	298(12)	425(31)	142(30)		400(8)	256(12)	83(14)	
Khulna	394(16)	163(12)	65(14)		743(14)	271(13)	70(12)	
Mymensingh	243(10)	96(7)	30(6)		691(13)	271(13)	66(11)	
Rajshahi	369(15)	123(9)	46(10)		745(14)	255(12)	75(13)	
Rangpur	327(13)	117(8)	38(8)		808(16)	245(11)	47(8)	
Sylhet	303(12)	119(9)	40(8)		624(12)	282(13)	81(14)	
**Hypertension**								
No	1967(78)	1034(75)	273(58)	**80.643 (<0.01)**	3984(77)	1640(77)	357(61)	**76.271 (<0.01)**
Yes	569(22)	347(25)	198(42)		1193(23)	498(23)	230(39)	
**BMI**								
Underweight	376(15)	147(11)	35(8)	**87.431 (<0.01)**	1070(21)	391(19)	67(12)	**91.688 (<0.01)**
Normal	1024(41)	486(35)	126(27)		2323(45)	898(43)	206(36)	
Overweight	1114(44)	740(54)	302(65)		1726(34)	822(39)	303(53)	
**Physical activities**								
**Yes**	750(30)	350(25)	82(17)	**32.896 (<0.01)**	2400(47)	883(41)	193(33)	**46.357 (<0.01)**
**No**	1776(70)	1025(75)	389(83)		2761(53)	1247(59)	389(67)	
**Wealth status**								
Poorest	300(12)	64(5)	18(4)	**227.21 (<0.01)**	1396(27)	514(24)	99(17)	**184.27 (<0.01)**
Poorer	276(11)	84(6)	17(4)		1365(26)	488(23)	106(18)	
Middle	444(17)	176(13)	48(10)		1189(23)	457(21)	121(21)	
Richer	654(26)	357(26)	109(23)		790(15)	382(18)	125(21)	
Richest	865(34)	700(51)	473(59)		441(9)	297(14)	136(23)	

Table 3. Ordinal logistic regression table for urban and rural areas

Variables		Urban				Rural			
				95% C.I				95% C.I	
		AOR	p-value	Lower	Upper	AOR	p-value	Lower	Upper
**Gender**									
	Male (ref)	-	-						
	Female	1.063	0.344	0.937	1.206	0.959	0.408	0.869	1.059
**Age**									
	Youth (ref)	-	-						
	Prime working	1.350	**<0.001**	1.143	1.597	1.561	**<0.001**	**1.358**	**1.796**
	Mature working	1.882	**<0.001**	1.462	2.421	1.874	**<0.001**	**1.545**	**2.273**
	Older	1.645	**<0.001**	1.251	2.162	1.523	**<0.001**	**1.237**	**1.875**
**Education level**									
	No education (ref)	-	-						
	Primary	1.004	0.966	0.835	1.208	1.099	0.139	0.969	1.248
	Secondary	0.828	0.056	0.683	1.005	0.992	0.911	0.861	1.143
	Higher	0.673	**<0.001**	0.539	0.839	0.805	**0.028**	**0.663**	**0.977**
**Caffeinated drink**		-	-						
	No (ref)								
	Yes	1.093	0.416	0.881	1.354	1.243	**0.025**	**1.026**	**1.501**
**Smoking status**									
	No (ref)	-	-						
	Yes	0.977	0.810	0.806	1.182	0.985	0.827	0.864	1.123
**BMI**									
	Underweight (ref)	-	-						
	Normal	1.093	0.400	0.889	1.348	1.098	0.166	0.962	1.254
	Overweight	1.399	**0.002**	1.137	1.727	1.318	**<0.001**	**1.143**	**1.521**
**Wealth status**									
	Poorest (ref)	-	-						
	Poorer	1.257	0.186	0.896	1.768	0.973	0.698	0.849	1.116
	Middle	1.761	**<0.001**	1.311	2.382	1.072	0.336	0.9302	1.236
	Richer	2.647	**<0.001**	2.009	3.519	1.397	**<0.001**	**1.196**	**1.632**
	Richest	4.165	**<0.001**	3.156	5.549	2.152	**<0.001**	**1.800**	**2.572**
**Physical activity**									
	Yes (ref)	-	-						
	No	1.092	0.253	0.939	1.269	1.294	**<0.001**	**1.169**	**1.433**
**Hypertension**									
	No (ref)	-	-						
	Yes	1.256	**0.002**	1.086	1.452	1.094	0.125	0.975	1.226

Note. AOR. Adjusted odds ratio; BMI. Body mass index

Figure

Click here to access/download;Figure;Figures.docx

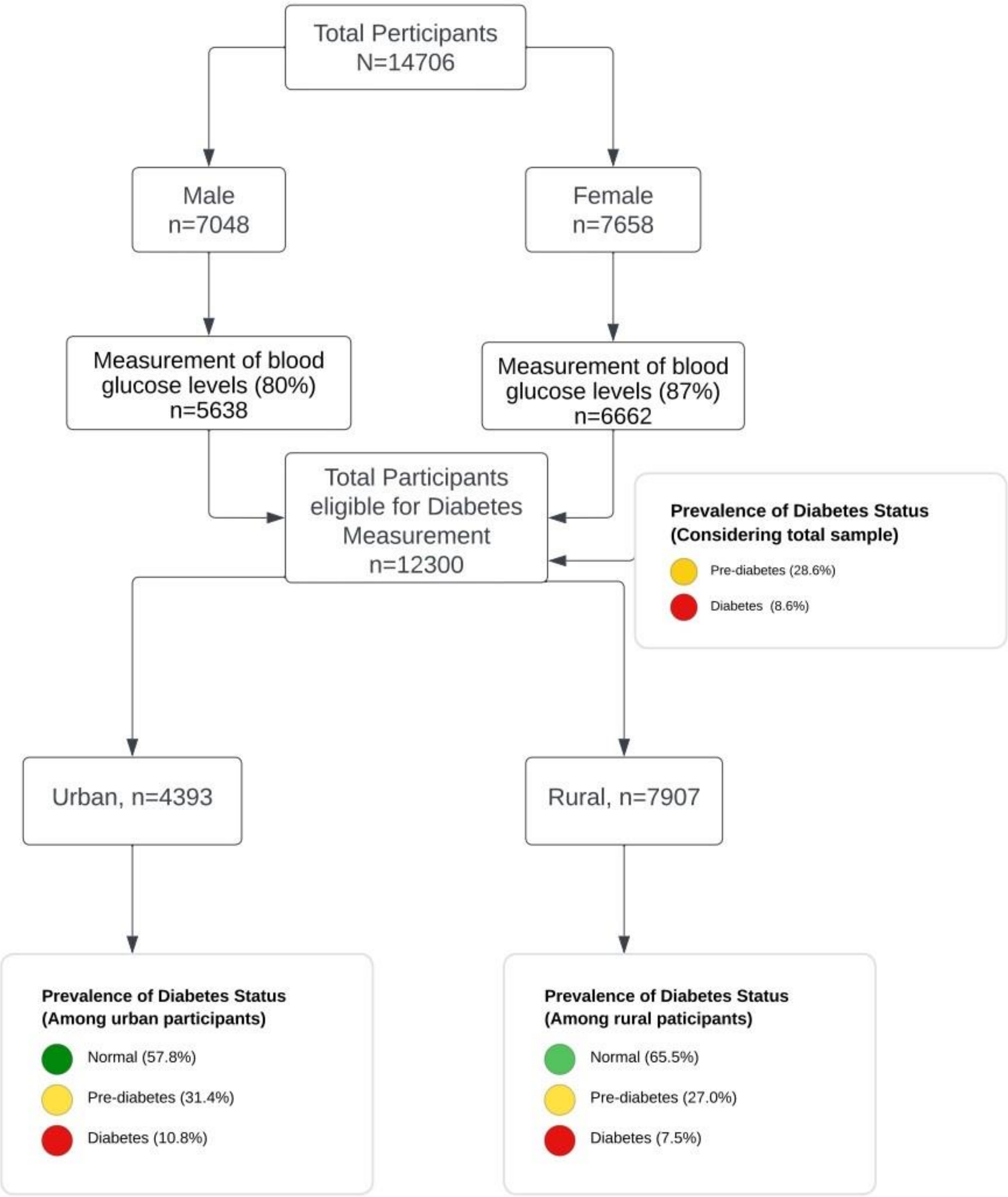

Figure 1: Flow chart showing sample selection procedure
