# Supplementary material for: Rural and urban differences in the prevalence and determinants of Type-2 diabetes in Bangladesh
Source: PLoS One. 2024 Apr 11;19(4):e0298071. doi: 10.1371/journal.pone.0298071 (PMC11008877; doi:10.1371/journal.pone.0298071)
Supplement: Attachment — Submitted filename: Response to Reviewer comments (1).docx [file pone.0298071.s002.docx]

Dear Editor (s) and reviewers,

I am delighted to inform you that I, hereby, submit our revised manuscript titled ‘**Rural and Urban Differences in the Prevalence of Type-2 Diabetes and the Associated Risk Factors: Analysis of a Population-Based Nation-wide Cross-sectional Survey in Bangladesh’** in your journal for possible publication.

We thank you for giving us the opportunity to revise and resubmit the manuscript. We greatly appreciate the valuable comments from the editor/reviewers and have modified the enclosed manuscript accordingly. In this response letter enclosed, we quote all comments and clarify what we have done in response to each of them. Below, we include reviewers’ comments and our responses to each of the comments.

All changes are highlighted in yellow colour. We hope that our revisions have adequately addressed editor/reviewers’ comments/concerns. We are confident that the revisions have improved the quality of our manuscript. We look forward to hearing from you with a final decision regarding the acceptance of our manuscript.

Regards

Ashis Talukder

**The point-by-point response of the reviewer’s comments are mentioned below:**

**Reviewer #1:** The authors have addressed the comments raised during the review process and the paper is now in good shape. I now have only minor suggestions, especially in the discussion section and the table.

**Policy implications:** redundant information from the opening paragraph of the discussion. Please remove.

**Policy implications:** Maybe you could start with policy implications which are specific to your findings and not trying to mix-up what the study does not suggest. You could then close the paragraph with a more recommendable statement considering the wider policy environment.

**Response:** Thank you for your suggestions. We have revised the "Policy Implications" section in our manuscript in accordance with the specific findings obtained from our study. Kindly take a moment to review the updated "Policy Implications" section in our revised manuscript.

Table: Renaming will be required to describe the caption of the tables. Just mentioning frequency distribution or ordinary logistic regression would not provide enough information to the readers. For instance, table 1 might be renamed as Characteristics of the study participants. Table 3 might be predictors of T2DM across urban and rural areas, the statistical method used could be mentioned below the table. Please go through other literature as a reference.

**Response:** Thank you for providing feedback. We have incorporated your suggestions and revised the captions of the tables in our manuscript. Kindly review the updated captions to ensure they align with your recommendations.

**Reviewer #3:** The authors of the manuscript entitled "Rural and Urban Differences in the Prevalence and Determinants of Type-2 Diabetes in Bangladesh "have performed all comments. Thus, the manuscript can be accepted for publication.

**Response:** Thank you for your recommendation.
